# HBV and HCV Infection in Children and Adolescents

**DOI:** 10.3390/vaccines11020330

**Published:** 2023-02-01

**Authors:** Maria Pokorska-Śpiewak, Magdalena Marczyńska

**Affiliations:** 1 Department of Children’s Infectious Diseases, Medical University of Warsaw, 01-201 Warsaw, Poland; 2 Regional Hospital of Infectious Diseases in Warsaw, ul. Wolska 37, 01-201 Warsaw, Poland

## 1. Introduction

Hepatitis B (HBV) and C (HCV) infections are the major causes of chronic liver disease and are associated with significant morbidity and mortality. Thus, hepatitis B and C have been recognized by the World Health Organization (WHO) as one of the major global public health problems. In 2016, the WHO initiated an ambitious plan to eliminate viral hepatitis as a public health threat by 2030. Unfortunately, in the global strategy updated in 2022, the WHO stated that although significant progress had been made, most global health targets for 2020 had been missed [1]. The COVID-19 pandemic has further hampered the elimination strategy. As a result of chronic liver disease and cancer, hepatitis B and C together continue to cause 1.1 million deaths per year. Access to birth-dose hepatitis B vaccine remains low in many low- and middle-income countries. In addition, nearly 80% of people with hepatitis B or C virus remain undiagnosed, and affordable treatments are not being implemented [1]. The latest WHO global strategy established the following coverage targets to be achieved by 2030: the diagnosis of 90% of people living with HBV and HCV, as well as the treatment of 80% of patients eligible for therapy [1]. 

Compared to adult patients, little attention has been given to treating children and adolescents living with chronic hepatitis C or hepatitis B. Thus, important gaps in the evidence on the management, diagnosis, and treatment of pediatric HBV- and HCV-infected patients exist. Their access to modern therapies remains limited. This editorial aims to present the updated information on epidemiological issues, diagnostics, and treatment in children and adolescents with hepatitis B and C, which will be described in detail within the articles included in this Special Issue of *Vaccines*.

### 1.1. Hepatitis B

The estimated global prevalence of HBV infection in children aged 5 years or younger is 1.3% [2]. Despite the availability of highly effective universal immunizations, there are almost 2 million annual new HBV infections in children in this age group, mostly through mother-to-child transmission during birth or pregnancy [1,2]. This is of particular importance, as the risk of chronic (over 6 months) HBV infection is highest (90%) in cases of vertical infection in neonates or infants, compared to 30% in young children, and <5% for older children and adults [2,3]. HBV infection in pediatric patients is usually asymptomatic and characterized by a high-replication, low-inflammation phase, with detectable HBeAg and HBsAg in serum, and normal or slightly increased levels of aminotransferases. This phase may last for several decades [2]. The natural history of HBV infection is complex and dynamic, with disease progression to significant liver fibrosis, cirrhosis, and hepatocellular carcinoma (HCC), which are rarely observed in pediatric patients. Cirrhosis has been reported in 1–5% of HBeAg-positive children [2]. 

The main goal of antiviral treatment of hepatitis B includes the effective and sustained suppression of HBV replication, which consequently reduces the risk of disease progression to cirrhosis and HCC [4]. A “complete cure” of HBV infection requires not only HBsAg loss and undetectable serum HBV DNA, but also the clearance of covalently closed circular DNA (ccc-DNA); otherwise, only a “functional cure” may be achieved [4,5]. Current guidelines for HBV therapy in children are available from ESPGHAN and AASLD [6,7]. The decision to start treatment in pediatric patients with hepatitis B is based on the combined assessment of stage of liver disease, HBV DNA viral load, alanine aminotransferase concentration, and HBeAg status. In each case, the possible benefits of treatment need to be analyzed against the disadvantages of initiating long-term therapy [2]. Family history of HCC, HIV coinfection, or a coincidence of other liver disease or immunosuppressive therapy may support a decision for earlier antiviral treatment [2,4]. Some of the antiviral drugs approved for children and adolescents with chronic HBV infection include interferon alfa-2b, peginterferon alfa-2a, and nucleoside/nucleotide analogue therapies with lamivudine, entecavir, adefovir, tenofovir disoproxil fumarate, and tenofovir alafenamide [4,6,7]. It has been estimated that with the use of available therapies, HBsAg loss and HBsAg seroconversion to anti-HBs may be achieved in only 1-6% of children [8]. New HBV treatment strategies aimed at eliminating all replicative intermediates, including ccc-DNA in the nucleus, are required and are being researched, with the potential to transform chronic HBV infection into a curable disease [2]. In addition, universal access to hepatitis B birth-dose vaccines and improved testing of pregnant women for preventing vertical (mother-to-child) transmission of hepatitis B are needed to meet the WHO goals [1].

### 1.2. Hepatitis C

According to the recent epidemiological reports, there are over 3.2 million children living with HCV worldwide, most of them remaining undiagnosed [9]. However, most national hepatitis C policies lack specific recommendations for HCV testing in children and adolescents [10]. Chronic HCV infection in pediatric patients is usually considered a mild disease with only slow progression of liver disease. The risk of cirrhosis in children and adolescents with chronic hepatitis C is usually estimated at 1 to 2% [11,12,13,14,15]. However, recent observational studies demonstrated that a significantly higher proportion of pediatric patients may develop advanced liver disease resulting from HCV infection [16,17,18]. Analysis of the outcomes in 1049 patients infected with HCV during early childhood revealed that serious liver disease was developed in 32% of cases at a median of 33 years after infection, irrespective of the mode of infection [18]. The risk of HCC was 5%, the incidence of liver transplant 4%, and death 3% [18]. In addition, it was demonstrated that HCV infection in adolescents leads to a decreased health-related quality of life, poor social functioning, and reduced intelligence and memory testing [19]. 

To prevent these consequences of chronic HCV infection, early anti-HCV treatment should be implemented irrespective of HCV genotype, previous ineffective antiviral treatment, or the extent of liver fibrosis. New, highly effective and safe interferon-free therapies based on direct-acting antivirals (DAAs) have significantly changed the natural history of the disease, providing a chance for HCV eradication. The first DAAs were approved for use in children aged 12–17 years by the European Medical Agency and US Food and Drug Administration in 2017 [20]. Currently, several fixed-dose DAA combinations are available for use in pediatric patients aged 3 years and older, including the pangetotypic DAAs sofosbuvir/velpatasvir and glecaprevir/pibrentasvir. So far, more than 2000 pediatric patients worldwide have received treatment with DAAs, with an excellent efficacy and a very good safety profile [21]. In a recent systematic review with meta-analysis on the efficacy and safety of DAAs in children and adolescents, it was demonstrated that among patients receiving all doses of treatment, 100% of cases reached sustained virologic response (SVR) [21]. However, due to the prohibitive prices of DAAs, only a few countries have included recommendations for the treatment of pediatric patients infected with HCV in their national policies and strategies [22,23]. Thus, only a small proportion of children and adolescents have been treated, mainly during clinical trials. 

## 2. Conclusions

In order to achieve the WHO goals to eliminate chronic viral hepatitis B and C, children and adolescents should be involved in both screening and therapeutic programs. Modern anti-HCV therapies are safe and effective for pediatric patients. Therapeutic options for HBV treatment are limited and new strategies are needed to transform chronic HBV infection into a curable disease. This issue will be discussed in detail in the subsequent articles published in this Special Issue of *Vaccines*.
